# The Combination of Cigarette Smoking and Alcohol Consumption Synergistically Increases Reactive Carbonyl Species in Human Male Plasma

**DOI:** 10.3390/ijms22169043

**Published:** 2021-08-22

**Authors:** Kanae Mure, Susumu Tomono, Minae Mure, Mano Horinaka, Michihiro Mutoh, Toshiyuki Sakai, Hideki Ishikawa, Keiji Wakabayashi

**Affiliations:** 1 Department of Public Health, Wakayama Medical University School of Medicine, Wakayama 641-8509, Japan; 2 Graduate Division of Nutritional and Environmental Sciences, University of Shizuoka, Shizuoka 422-8526, Japan; tomono@aichi-med-u.ac.jp (S.T.); kwakabayashi@u-shizuoka-ken.ac.jp (K.W.); 3 Department of Microbiology and Immunology, Aichi Medical University School of Medicine, Nagakute 480-1195, Japan; 4 Department of Chemistry, The University of Kansas, Lawrence, KS 66045, USA; mmure@ku.edu; 5 Department of Drug Discovery Medicine, Kyoto Prefectural University of Medicine, Kyoto 602-8566, Japan; m-hori@koto.kpu-m.ac.jp (M.H.); tsakai@koto.kpu-m.ac.jp (T.S.); 6 Department of Molecular-Targeting Prevention, Kyoto Prefectural University of Medicine, Kyoto 602-8566, Japan; mimutoh@ncc.go.jp (M.M.); cancer@gol.com (H.I.)

**Keywords:** reactive carbonyl species, cigarette smoking, alcohol drinking, human plasma

## Abstract

Cigarette smoking and alcohol consumption are major risk factors for lifestyle-related diseases. Although it has been reported that the combination of these habits worsens risks, the underlying mechanism remains elusive. Reactive carbonyl species (RCS) cause chemical modifications of biological molecules, leading to alterations in cellular signaling pathways, and total RCS levels have been used as a lipid peroxidation marker linked to lifestyle-related diseases. In this study, at least 41 types of RCS were identified in the lipophilic fraction of plasma samples from 40 subjects using liquid chromatography/electrospray ionization tandem mass spectrometry (LC/ESI-MS/MS). Higher levels of 10 alkanals, 5 *trans*-2-alkenals, 1 *cis*-4-alkenal, and 3 alkadienals were detected in the smoking/drinking group (*N* = 10) as compared to those with either habit (*N* = 10 each) or without both habits (*N* = 10) in the analysis of covariances adjusted for age and BMI. The levels of 3 alkanals, 1 *trans*-2-alkenal, 1 alkadienal, and 1 4-hydroxy-2-alkenal in the smoking/drinking group were significantly higher than those in the no-smoking/drinking and no-smoking/no-drinking groups. These results strongly indicate that the combination of cigarette smoking and alcohol drinking synergistically increases the level and variety of RCS in the circulating blood, and may further jeopardize cellular function.

## 1. Introduction

Smoking and alcohol consumption have been reported as major risk factors for cancers, cardiovascular diseases (CVD), and other lifestyle-related diseases. According to the World Health Organization (WHO), tobacco use, including cigarette smoking, causes more than 7 million deaths worldwide each year [1]. WHO also estimates alcohol consumption contributes to 3 million deaths each year globally as well as to the disabilities and the poor health of millions of people worldwide [2]. It has been reported that the combination of smoking and alcohol drinking worsens the development and progression of various cancers and CVD [3,4,5]; however, the underlying mechanism remains elusive.

More than 4000 chemicals have been identified in cigarette smoke; about 250 of them are known to be health hazards, and more than 50 are carcinogens [6]. Cigarette smoke also contains highly reactive free radicals which promote reactive oxygen species (ROS) production [7,8]. ROS cause alterations to nucleic acids and proteins, and generate reactive carbonyl species (RCS) via lipid peroxidation [9]. Various types of RCS have been detected in cigarette mainstream smoke, alcoholic beverages, and human biological samples (Table S1) [10,11,12,13,14,15,16,17,18].

RCS are mostly produced by the autooxidation of unsaturated fatty acids in plants, cooking oils, and high-fat foods, but are also produced by oxidation of the essential components of cellular membranes (e.g., sugars, amino acids, polyamines, and unsaturated fatty acids) as well as peroxidation of lipids (e.g., phospholipids, triacylglycerols, cholesterol, and cholesteryl esters) via enzymatic or non-enzymatic processes (Table S1) [19,20,21,22,23,24]. RCS are relatively stable and have longer half-lives than ROS and reactive nitrogen species; they can cause or exacerbate damaging effects with regard to nucleic acids, proteins, cell membrane, and mitochondrial functions [25]. Consequently, RCS contribute to the development and progression of various diseases such as cancer, CVD, and the long-term complications of diabetes, chronic obstructive pulmonary disease, and neurodegenerative diseases [26,27,28,29,30].

The combination of smoking and alcohol drinking is anticipated to have synergetic effects in inducing RCS production, causing damage to endogenous cellular components and ultimately resulting in deleterious effects on human health. Although the total RCS level has been widely used as a biomarker for lipid peroxidation with regard to the development of several diseases such as cancer and diabetes mellitus, a detailed identification of RCS related to cigarette smoking in conjunction with alcohol drinking has not been studied to date.

We developed a sensitive and specific analytical method for the comprehensive analysis of RCS in biological samples using liquid chromatography/electrospray ionization tandem mass spectrometry (LC/ESI-MS/MS) [31]. Using this method, we elucidated the mechanisms of chemoprevention in tumors and cognitive decline in mice [32,33,34]. In this study, we applied our method to identify a variety of RCS in human plasma samples. To our knowledge, this is the first study characterizing RCS in plasma samples taken from human subjects with the consideration of their smoking and alcohol consumption habits.

## 2. Results

### 2.1. Characteristics of Subjects and Bubble Charts of RCS Detected in Human Plasma Samples

**Figure 1:**
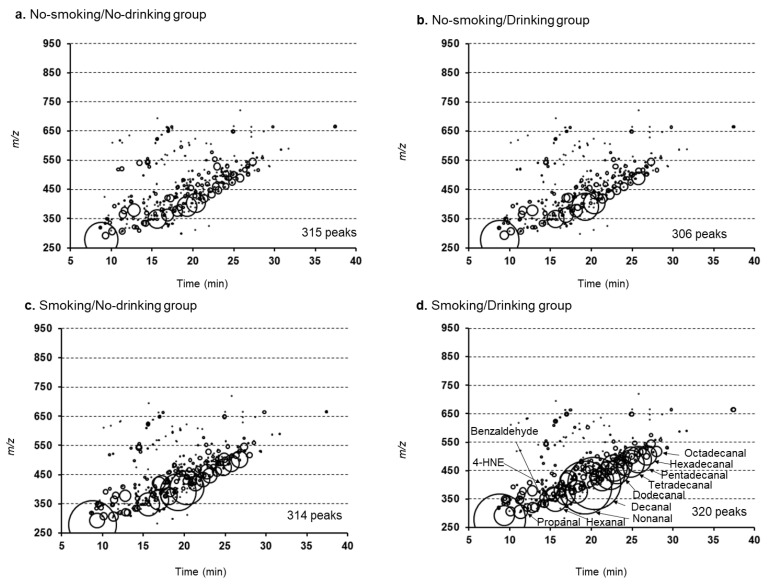
Bubble charts of reactive carbonyl species (RCS) in the plasma samples. (**a**) No-smoking and no-drinking group; (**b**) No-smoking and drinking group; (**c**) Smoking and no-drinking group; and (**d**) Smoking and drinking group.

The characteristics of subjects are shown in Table 1. Figure 1 shows bubble charts of lipophilic RCS detected in plasma samples from the no-smoking/no-drinking group (a), the no-smoking/drinking group (b), the smoking/no-drinking group (c), and the smoking/drinking group (d). The free RCS (open circles) were plotted as a function of their retention times (the horizontal axis) and *m*/*z* values (the vertical axis). The area of the circle represents the intensity of the peak of RCS detected relative to that of the internal standard. In total, 315, 306, 314, and 320 peaks were detected in the plasma samples taken from the no-smoking/no-drinking group, no-smoking/drinking group, smoking/no-drinking group, and smoking/drinking group, respectively (based on an average of 10 subjects). We eliminated redundant peaks from the spectra and included the spike noise and artifactual dansyl hydrazine derivatives. Most of the RCS identified were within *m*/*z* values between 250 and 650. A series of aldehydes with small molecular weights, including glyoxal (t_R_: 10.1 min, *m*/*z* 306) and propanal (t_R_: 11.1 min, *m*/*z* 306), are shown in the bottom left corner of Figure 1. Several large circles also appear side by side diagonally in the center of Figure 1. As the retention times increased, the molecular weights of these peaks increased in *m*/*z* increments of 14. By comparing these peaks with those of the authentic RCS samples, they were identified as fatty acid-derived aldehydes such as hexanal (t_R_: 15.6 min, *m*/*z* 348), decanal (t_R_: 20.4 min, *m*/*z* 404), and hexadecanal (t_R_: 25.8 min, *m*/*z* 488). The most abundant peak detected at a retention time of 19.3 min with an *m*/*z* value of 390 was identified as nonanal. In addition, 4-hydroxy-2-nonenal (t_R_: 14.0 min, *m*/*z* 404) was also detected with low abundance.

### 2.2. Heatmap of the Levels of RCS Detected in the Plasma Samples 

**Figure 2:**
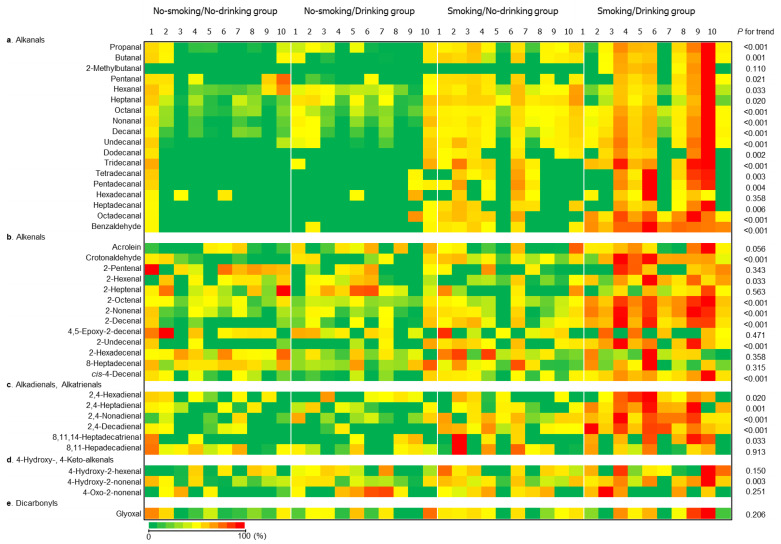
Heat map of the levels of reactive carbonyl species (RCS) in the individual subjects in the present study. Green color cells indicate that RCS was not detected in the plasma samples. The maximum levels of each RCS are colored red.

The levels of RCS detected in the plasma samples from each subject are shown as a heat map in Figure 2, and the chemical structures of the identified RCS are summarized in Table S1. The highest levels of RCS (in red) were mostly detected in the smoking/drinking group. Significant trends were observed in most of the RCS, especially for alkanals (e.g., propanal, octanal, nonanal, decanal, undecanal, tridecanal, and octadecanal), and an aromatic alkanal (benzaldehyde), *trans*-2-alkenals (crotonaldehyde, 2-octenal, 2-nonenal, 2-decenal and 2-undecenal), a *cis*-4-alkenal (*cis*-4-decenal), and alkadienals (2,4-nonadienal and 2,4-decadienal) exhibited the greatest significant trend (*p* < 0.001).

### 2.3. The Relative Levels of RCS Detected in the Plasma Samples of Each Group Compared to Those of the No-Smoking/No-Drinking Group

**Figure 3:**
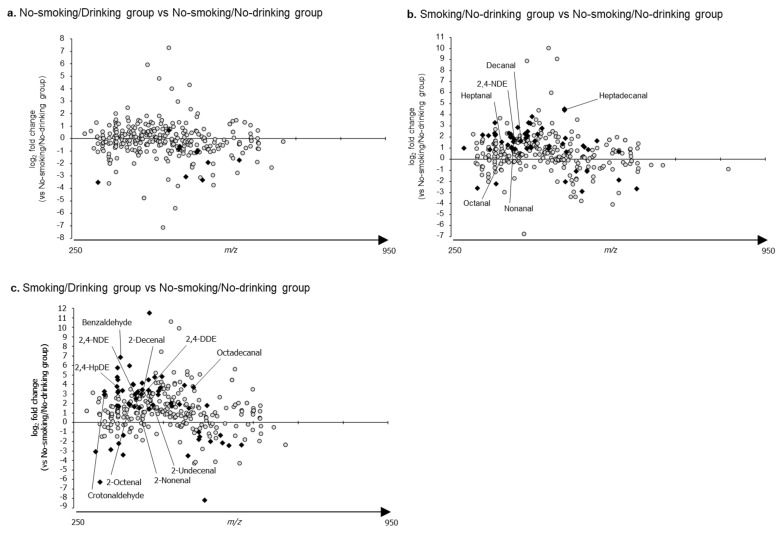
The profiles of reactive carbonyl species (RCS) detected in the plasma samples of each group as compared to the no-smoking and no-drinking group. (**a**) No-smoking and drinking group; (**b**) Smoking and no-drinking group; (**c**) Smoking and drinking group. The gray circles indicate the levels of each RCS, and closed diamonds indicate the levels of RCS that showed significant differences as compared to those of the no-smoking and no-drinking group (*p* < 0.05) in 1-way analyses variance with post hoc Bonferroni analysis.

The relative levels of the RCS detected in the plasma samples of each group were compared to those of the no-smoking/no-drinking group (Figure 3). Overall, 10 out of 314 peaks were detected at more elevated levels in the no-smoking/drinking group as compared to the in no-smoking/no-drinking group (Figure 3a). Significant differences in 54 out of 314 peaks (e.g., heptanal, 2,4-nonadienal, nonanal, octanal, decenal, and heptadecanal) were observed for the smoking/no-drinking group as compared to the no-smoking/no-drinking group (Figure 3b). Similarly, significant differences in 58 out of 320 peaks (e.g., octadecanal, benzaldehyde, crotonaldehyde, 2-octenal, 2-nonenal, 2-decenal, 2-undecenal, 2,4-nonadienal, and 2,4-decadienal) were observed in the smoking/drinking group as compared to the no-smoking/no-drinking group (Figure 3c). There were some compounds detected at lower levels in all groups as compared to those in the no-smoking/no-drinking group; however, we were not able to identify them due to their low abundance. The levels of identified RCS in each group and the comparisons among groups are shown in Table S2.

### 2.4. Body Mass Index, Alcohol Drinking, and Cigarette Smoking Are Associated with RCS Levels

In the multivariate regression analyses, body mass index (BMI) was significantly associated with ≤ C_12_ alkanals in addition to heptadecanal, whereas no significant associations were seen with *trans*-alkenals, alkadienals, and 4-hydroxy-2-alkenal, apart from acrolein, *cis*-4-decenal, and glyoxal (Table S3). Age was not significantly associated with the RCS identified in this study except 2-hexanal. Alcohol drinking was significantly associated with benzaldehyde, 2-octenal, and 2-nonenal (*p* < 0.001). Alcohol drinking was also associated with a total of 11 alkanals (propanal, 2-methylbutanal, octanal, nonanal, decanal, undecanal, dodecanal, tridecanal, tetradecanal, pentadecanal, and octadecanal), a total of 4 *trans*-alkenals (acrolein, 2-hexenal, 2-decenal, and 2-undecenal), 1 *cis*-4-alkenal (*cis*-4-decenal), 3 alkadienals (2,4-hexadienal, 2,4-heptadienal, and 2,4-decadienal), and 1 4-hydroxy-alkenal (4-hydroxy-2-nonenal). Alcohol drinking was significantly associated with a reduction of 2-hexadecenal. Smoking was significantly associated with 4 alkanals (octanal, nonanal, decanal, and octadecanal), 1 aromatic alkanal (benzaldehyde), 4 *trans*-alkenals (crotonaldehyde, 2-octenal, 2-nonenal, and 2-decenal), and 2 alkadienals (2,4-heptadienal and 2,4-decadienal) (*p* < 0.001). Smoking was also significantly associated with a total of 10 alkanals (propanal, butanal, hexanal, heptanal, undecanal, dodecanal, tridecanal, tetradecanal, pentadecanal, and heptadecanal), 2 *trans*-alkenals (2-hexenal and 2-undecenal), 1 *cis*-4-alkenal (*cis*-4-decenal), 2 alkadienals (2,4-hexadienal and 2,4-nonadienal), 1 alkatrienal (8,11,14-heptadecatrienal), and 1 4-hydroxy-alkenal (4-hydroxy-2-nonenal).

### 2.5. Smoking and Drinking Synergetically Produce RCS

**Figure 4:**
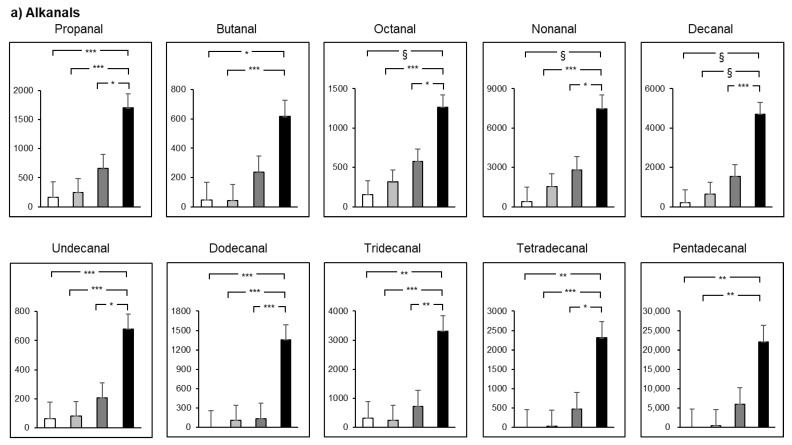

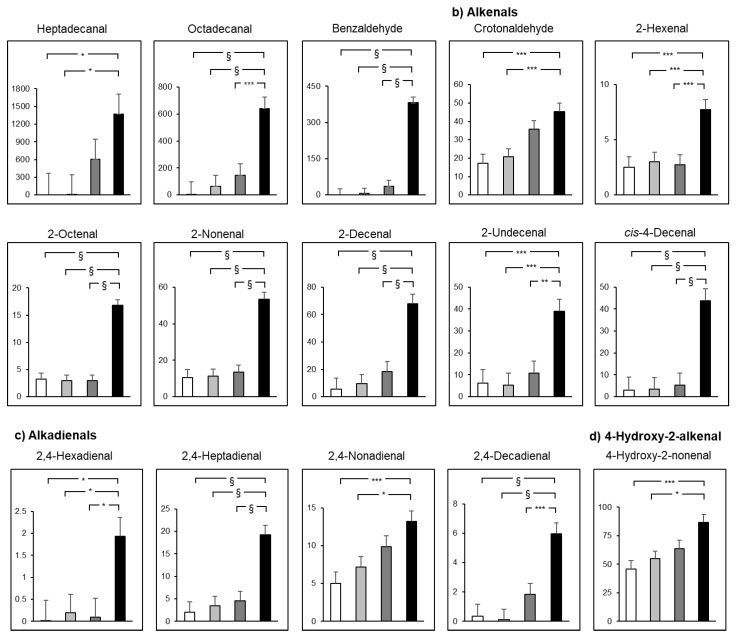
The levels (pmol/mL) of each reactive carbonyl species (RCS) were compared among each group (**a**–**d**). Open bars indicate RCS in the no-smoking and no-drinking group. Light gray bars indicate RCS in the no-smoking and drinking group. Dark gray bars indicate RCS in the smoking and no-drinking group. Solid bars indicate RCS in the smoking and alcohol-drinking group. Differences were analyzed by 1-way analysis covariance (ANCOVA) with post hoc Tukey analysis. The estimated means and errors are shown for each group. *: *p* < 0.05. **: *p* < 0.01, ***: *p* < 0.005, §: *p* < 0.001.

In the 1-way analysis covariance (ANCOVA) adjusted for age and BMI, a total of 12 alkanals (propanal, butanal, octanal, nonanal, decanal, undecanal, dodecanal, tridecanal, tetradecanal, pentadecanal, heptadecanal, and octadecanal) and 1 aromatic alkanal (benzaldehyde) showed significant differences among groups (Figure 4a). The levels of propanal, octanal, nonanal, decanal, undecanal, dodecanal, tridecanal, tetradecanal, octadecanal, and benzaldehyde in the smoking/drinking group were at significantly higher levels compared to the other groups. The levels of butanal, pentadecanal, and heptadecanal in the smoking/drinking group were significantly higher than those in the no-smoking/no-drinking and no-smoking/drinking group. A total of 6 *trans*-2-alkenals and 1 *cis*-4-alkenal (crotonaldehyde, 2-hexenal, 2-octenal, 2-nonenal, 2-decenal, 2-undecenal, and *cis*-4-decenal) showed significant differences among groups (Figure 4b). The levels of 2-hexenal, 2-octenal, 2-nonenal, 2-decenal, 2-undecenal, and *cis*-4-decenal were at significantly higher levels in the smoking/drinking group. The level of crotonaldehyde in the smoking/drinking group was significantly higher than in the no-smoking/no-drinking and no-smoking/drinking group. A total of four alkadienals (2,4-hexadienal, 2,4-heptadienal, 2,4-nonadienal, and 2,4-decadienal) showed significant differences among groups (Figure 4c). The levels of 2,4-hexadienal, 2,4-heptadienal, and 2,4-decadienal were significantly at the highest levels in the smoking/drinking group. The level of 2,4-nonadienal in the smoking/drinking group was significantly higher than in the no-smoking/no-drinking and no-smoking/drinking group. As for 4-hydroxy-2-alkenal, the level of 4-hydroxy-2-nonenal in the smoking/drinking group was significantly higher as compared to the no-smoking/no-drinking and no-smoking/drinking group (Figure 4d).

## 3. Discussion

In this study, we successfully identified a wide range of lipophilic RCS in chloroform/methanol extractable fractions of human plasma samples by dansyl hidrazine-derivatization followed by LC/ESI-MS/MS analysis in a selected reaction mode that we developed previously. The combination of smoking and alcohol drinking clearly showed significant synergistic effects on the plasma level of RCS, especially with regard to *trans*-2-alkenals (2-hexenal, 2-octenal, 2-nonenal, 2-decenal, and 2-undecenal), *cis*-4-alkenal (*cis*-4-decenal), alkadienals (2,4-hexadienal, 2,4-heptadienal, and 2,4-decadienal), alkanals (propanal, octanal, nonanal, decanal, undecanal, dodecanal, tridecanal, tetradecanal, and octadecanal), and an aromatic alkanal (benzaldehyde).

Alkanals (≤C_10_) and alkenals (≤C_6_) have been detected in cigarette smoke (Table S1) [10,11,12,13], as they are contained in tobacco leaves and are also used as flavor additives for cigarettes. Thus, there is no surprise that higher levels of RCS were detected in the smoking/no-drinking group as compared to the no-smoking/no-drinking group in this study. RCS such as acrolein, nonanal, and 2-nonenal have been detected in alcoholic beverages [17,18], and were also identified in the no-smoking/drinking group in the multivariate regression analysis in this study. However, no significant differences were detected in the no-smoking/drinking group as compared to the no-smoking/no-drinking group, whereas significant synergistic effects of drinking and smoking were observed in ANCOVA. Excessive alcohol consumption has been known to increase the level of free iron in the cell and promote ROS production, leading to RCS production. Since RCS are known to be downstream products of ROS [9], it is highly likely that ROS induced by alcohol drinking triggers further RCS production in smokers.

As summarized in Supporting Table S1, in human urine samples some alkanals ≤ C_11_ as well as alkenals ≤ C_10_, 2,4-alkadienals, and 4-hydroxy-2-nonenal were detected in other studies, and 2-methylpropanal and nonanal were seen at statistically higher levels in the smoking groups as compared to the no-smoking groups [15]. A total of 19 aldehydes (alkanals: C_2_–C_10_, alkenals: C_3_–C_9_, and benzaldehyde) have also been detected in sera from healthy adults, although the analyses did not provide information on the smoking and drinking habits of the subjects [14]. It should be noted that these previous studies did not include the extraction step with an organic solvent, limiting the detection of more lipophilic (≥C_12_) aldehydes. By including the chloroform/methanol extraction step in this study we were able to identify more lipophilic aldehydes and observed the more pronounced synergistic effects of smoking and alcohol drinking for longer carbon chain (≥C_12_) alkanals.

Another novel finding in our study was the synergistic interaction of smoking and alcohol drinking that was seen for *trans*-2-alkenals (2-pentenal, 2-hexenal, 2-octenal, 2-nonenal, 2-decenal, and 2-undecenal), 2,4-alkadienals (2,4-heptadienal and 2,4-decadienal), and 4-hydroxy-2-alkenal (4-hydroxy-2-nonenal). It has been reported that those RCS play pro-inflammatory roles and promote cellular proliferation.

*trans*-2-Alkenals have been detected in oxidized low-density lipoprotein (LDL) as well as in oxidized high-density lipoprotein (HDL), along with 2,4-alkadienals and several alkanals [35,36]. The oxidized LDL stimulates macrophages to induce atherosclerosis, and oxidized HDL has also been shown not only to accelerate atherosclerosis but also to contribute to tumor progression [37,38].

**Figure 5:**
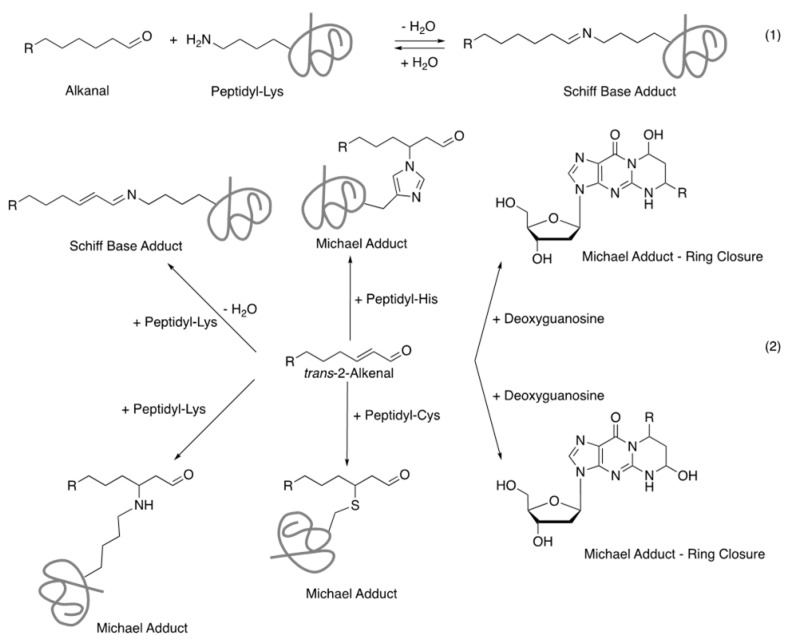
Adduct formation with aldehydes. Schiff base formation occurs between alkanal (1) or *trans*-2-alkenal (2) and the ϵ-amino group of the lysine side chain; Michael adducts formation occurs between *trans*-2-alkenal with nucleophilic amino acid side chains of peptides and proteins such as lysine, histidine, and cysteine residues, as well as nucleic acids (here deoxyguanosine is shown as an example) (2). *cis*-4-Decenal only forms Schiff base adducts. The Schiff base products are less stable than Michael adducts as they can easily undergo hydrolysis or further nucleophilic addition of nucleophilic amino acid side chains.

*trans*-2-Alkenals are known to form Schiff base adducts with lysine residues in peptides and proteins and Michael adducts with lysine, histidine, or cysteine residues in peptides, proteins, and nucleic acids, whereas alkanal and *cis*-4-decenal only form Schiff base adducts (Figure 5) [39,40]. Schiff base adducts are less toxic as they are easily broken down (subjected to hydrolysis), but Michael adducts are more stable (do not undergo hydrolysis). 4-Hydroxy-2-nonenal is also known to form both Schiff base and Michael adducts and that can initiate protein crosslinking [39]. 4-Hydroxy-2-nonenal has been shown to induce upregulation of proinflammatory factors and stimulate cellular signaling activity involving p38 mitogen-activated protein kinase (MAPK) and c-jun N-terminal kinase (JNK) [41]. They are also associated with CVD, diabetes, cancer, chronic kidney disease, and neurodegenerative diseases [42,43]. 2-Octenal, 2-hexenal, and 2,4-decadienal have shown to induce higher interleukin-1β (IL-1β) release, whereas alkanals such as hexanal, octanal, and decanal exhibited reduced release activities in human mononuclear cells [24,44]. IL-1β is known as a key component of proinflammatory cytokines and relates to the development and progression of many diseases (e.g., atherosclerosis, type II diabetes, rheumatoid arthritis, and neurogenerative diseases) [45,46].

2,4-Hexadienal and 2,4-decadienal have been shown to induce DNA-adduct formation and promote the proliferation of human cells and carcinogenesis in animals [47,48]. 2-Hexenal covalently modifies DNA and promotes cell proliferation and carcinogenesis in animals [49,50], and benzaldehyde induces DNA damage in human cells and is related to oral cancer [51,52]. Taken together, these previous findings strongly suggest that the *trans*-2-alkenals and alkadienals identified in this study could induce chronic inflammation and further trigger the aforementioned diseases. Further study is necessary to elucidate specific biological effects of the alkanals (decanal, tridecanal, and octadecanal) that exhibited significant synergistic effects from smoking and alcohol drinking in this study.

The combination of smoking and alcohol drinking has been shown to induce alterations in lipid profiles such as sphingomyelins and acyl-alkyl- and lyso-phosphatidylcholines [53]. Sphingomyelins and phosphatidylcholines are the essential components of cellular membranes and are the known targets for ROS in the generation of RCS [54]. The synergistic effects of smoking and alcohol drinking found in this study most likely correlate with the alteration of the lipid profiles.

There are some limitations in this study, for example the small number of subjects and a lack of information on inflammation and oxidative stress markers, as well as the dietary information of the participants. This study was aimed to evaluate the feasibility of our methodology for applications in future studies on a larger scale for the elucidation of the molecular mechanisms of RCS in the development of lifestyle-related diseases. In our larger scale study, we will include women, obtain necessary dietary information regarding the subjects, and examine markers for inflammation and oxidative stress in plasma samples.

To our knowledge, this is the first study to demonstrate that cigarette smoking and daily alcohol consumption synergistically elevate the levels of RCS in human plasma. Our results strongly indicate that these habits may have detrimental effects relating to the induction of cellular and organ dysfunction, leading to lifestyle-related diseases.

## 4. Materials and Methods

### 4.1. The Study Subjects and Sample Collection

The subjects included in this study were selected from participants of a nation-wide specific health checkup and health and welfare service for the elderly developed by the Japanese Ministry of Health, Labor and Welfare that was carried out in the rural area of Wakayama, Japan, in 2016. In total, 165 people (84 men, 81 women) aged 40–84 years (61.3 ± 10.5 years) were registered, and they provided informed consent prior to the study. Subjects completed a standardized self-administered questionnaire including lifestyle factors such as current smoking and alcohol drinking habits (Table S4). Height and weight were measured on site to calculate the BMI (weight/height^2^ (kg/m^2^)). Information on current smoking habits, the number of cigarettes per day, and total length of time of continuous smoking was provided. Participants who currently smoked daily (≥20 cigarettes/day) for ≥6-months were categorized into the smoking group, and those who had never smoked were categorized as the no-smoking group. Participants who drank alcohol beverages (≥20 g/day) every day were categorized into the drinking group, and those who never drink alcohol beverages were categorized as the no-drinking group. In total, 10 subjects were randomly selected for each of the 4 groups: the no-smoking/no-drinking group (a) (*N* = 10), the no-smoking/drinking group (b) (*N* = 10), the smoking/no-drinking group (c) (*N* = 10), and the smoking/drinking group (d) (*N* = 10). Among women, only 2 drank alcoholic beverages regularly (<10 g/day), and there were none who smoked cigarettes (Table S4). Consequently, women were excluded from the analyses to rule out sex bias among the 4 groups. Participants were asked to fast for at least 10 hours before the examination, according to the guidelines. Venous blood was collected in a heparinized vacuum blood collection tube; plasma was separated by centrifugation at 1500× *g* at 4 °C for 10 min and was immediately stored at −80 °C until the assay. The medication status of the subjects in this study is shown in Table S5.

### 4.2. Chemicals

All chemicals purchased were of analytical grade (ACS grade) and were used as received without any additional purification. Crotonaldehyde, dansyl hydrazine (DH), 2,4-decadienal, glyoxal, heptadecanal, hexadecanal, 2,4-nonadienal, octadecanal, pentadecanal, and tetradecanal were purchased from Tokyo Chemical Industry (Tokyo, Japan). *p*-Toluenesulfonic acid (*p*-TsOH), butanal, decanal, dodecanal, 2-hexenal, hexanal, 2-heptenal, heptanal, octanal, 2-nonenal, nonanal, pentanal, propanal, tridecanal, and undecanal were obtained from Sigma-Aldrich (St. Louis, MO, USA). 4,5-Epoxy-2-decenal, 4-hydroxy-2-hexenal, 4-hydroxy-2-nonenal, and 4-oxo-2-nonenal were purchased from the Cayman Chemical Company (Ann Arbor, MI, USA). All other chemicals were obtained from Wako Pure Chemical Industries (Osaka, Japan). 8-Heptadecenal, 8,11-heptadecadienal, and 8,11,14-heptadecatrienal were synthesized following the published method [31].

### 4.3. Extraction of RCS from Plasma and LC/ESI-MS/MS Analysis

The experimental details of the RCS extraction from plasma samples and LC/ESI-MS/MS analyses were described previously [31]. Briefly, 20 μL of plasma was mixed with 180 μL of 50 mM sodium phosphate buffer (pH 7.4) containing 0.5 mM EDTA and 20 μM butylated hydroxytoluene. The mixture was added to 400 μL of chloroform/methanol (2:1, *v*/*v*) solution containing *p*-benzyloxybenzaldehyde (20 pmol) as the internal standard, and centrifuged at 20,000× *g* for 10 min at 4 °C. The organic phase was removed and set aside. The aqueous phase and precipitates were mixed with another 400 μL of the chloroform/methanol solution, and the resulting mixture was centrifuged to isolate the organic phase. Then, 100 μL of acetonitrile containing 50 μg (0.19 μmol) of DH and 10 μg (0.06 μmol) of *p*-TsOH was added to the combined organic phase and the mixture was incubated for 4 hours at room temperature in the dark. The solvent was removed from the reaction mixture to dryness in vacuo to yield the corresponding DH-derivatized products. These products were then dissolved in 200 μL of acetonitrile, and 5 μL aliquots were injected into the LC/ESI-MS/MS system per run.

The RCS-DH derivatives were separated on a TSK-gel Super Octyl column (2.3 μm, 100 mm × 2.0 mm, TOSOH, Tokyo, Japan) connected to a Dionex UltiMate3000 system (Thermo Fisher Scientific, Waltham, MA, USA) and a TSQ Endura triple-stage quadrupole tandem mass spectrometer with a heated electrospray ionization source (Thermo Fisher Scientific). RCS-DH derivatives were eluted from the column with a programmed linear gradient: mobile phase A consisted of a 0.1% (*v*/*v*) solution of formic acid in MilliQ water, and mobile phase B consisted of a 0.1% (*v*/*v*) solution of formic acid in acetonitrile, changing from 20% B to 100% B in 10 min at a flow rate of 0.2 mL/min. The elution of RCS-DH was completed within 10 min. The column was then washed with 100% B for 10 min and re-equilibrated to 20% B for 10 min before the next sample was injected. The instrument parameters for the positive-ion mode were as follows: ionspray voltage, 3500 V; ion transfer tube temperature, 325 °C; vaporizer temperature, 275 °C; sheath gas flow at 35 arbitrary units; auxiliary gas flow at 10 arbitrary units; and collision energy at 40 V. The RCS-DH derivatives were detected using the selected reaction mode. Formaldehyde and acetaldehyde were excluded from the results because chloroform/methanol was used for extraction of RCS from plasma samples. Quality assurance/quality control (QA/QC) procedures were performed followed by same method described previously [31].

### 4.4. Statistical Analyses

A statistical analysis of detected peaks of RCS-DH derivatives was performed using GraphPad Prism 8 software (GraphPad Software, San Diego, CA, USA). All other statistical analyses were performed using STATA version 16 (STATA Corp, College Station, TX, USA). Differences among groups were analyzed by 1-way analysis of variance (ANOVA) with the Bonferroni post hoc test. The associations of age, BMI, alcohol drinking, and smoking habits with RCS levels were analyzed by multivariate regression analyses. Differences among groups were analyzed by a 1-way analysis of covariates (ANCOVA) adjusted for age and BMI with Tukey´s post hoc test. P for trends was also evaluated, and the level of significance was set as *p* < 0.05.

## Figures and Tables

**Table 1:** Characteristics of the subjects.

	No-Smoking/No-Drinking Group	No-Smoking/Drinking Group	Smoking/No-Drinking Group	Smoking/Drinking Group	P ^a^	P ^b^	P ^c^	P ^d^	P ^e^	P ^f^
*N*	10	10	10	10						
Age (years)	73.2 ± 7.6	63.2 ± 8.4	59.5 ± 10.8	59.6 ± 10.2	0.132	0.014	0.015	1.000	1.000	1.000
BMI (kg/m^2^) ^1^	22.9 ± 3.0	23.5 ± 3.0	24.4 ± 2.3	23.2 ± 2.7	1.000	1.000	1.000	1.000	1.000	1.000
Triglyceride (mg/dL)	89.2 ± 38.0	107.4 ± 55.0	126.6 ± 60.2	237.3 ± 215.4	1.000	1.000	0.045	1.000	0.106	0.246
HDL-C (mg/dL) ^2^	57.5 ± 16.0	59.8 ± 16.6	48.3 ± 9.6	55.4 ± 9.6	1.000	0.799	1.000	0.376	1.000	1.000
LDL-C (mg/dL) ^3^	105.2 ± 28.2	104.5 ± 31.5	137.8 ± 37.7	101.0 ± 25.9	1.000	0.150	1.000	0.133	1.000	0.073
Drinking habits ^4^	no	yes	no	yes						
Number of cigarettes/day	0	0	26 ± 7.0	24 ± 5.7						0.492
Brinkman index	0	0	1008 ± 355.7	882 ± 335.0						0.425

^1^ BMI: Body mass index; ^2^ HDL-C: high-density lipoprotein cholesterol; ^3^ LDL-C: low-density lipoprotein cholesterol; ^4^ Yes: daily alcohol consumption ≥ 20 g/day; P: 1-way analysis of variance with Bonferroni post hoc test; P ^a^: no-smoking/no-drinking group vs. no-smoking/drinking group; P ^b^: no-smoking/no-drinking group vs. smoking/no-drinking group; P ^c^: no-smoking/no-drinking group vs. smoking/drinking group; P ^d^: no-smoking/drinking group vs. smoking/no-drinking group; P ^e^: no-smoking/drinking group vs. smoking/drinking group; P ^f^: smoking/no-drinking group vs. smoking/drinking group.

## Data Availability

Data will not be made available, but the data that support the findings of this study are available on reasonable request to the corresponding author.

## Supplementary Materials

The following are available online at https://www.mdpi.com/article/10.3390/ijms22169043/s1, Table S1: Chemical characteristics of RCS identified in human plasma samples in this study, Table S2: Comparison of the levels of RCS in human plasma samples among each group, Table S3: Association of age, BMI, drinking, and smoking habits on RCS levels in plasma, Table S4: The basic characteristics of participants, Table S5: Medication status of subjects in this study.
